# Validity and reliability of the Greek translation of the Job Satisfaction Survey (JSS)

**DOI:** 10.1186/s40359-018-0241-4

**Published:** 2018-06-08

**Authors:** Andreas Tsounis, Pavlos Sarafis

**Affiliations:** 1 Aristotle University of Thessaloniki, School of Psychology, Thessaloniki, Greece; 2 Department of Nursing, School of Health Sciences, Cyprus University of Technology 15, Vragadinou Str, 3041 Limassol, Cyprus

**Keywords:** Job satisfaction, JSS questionnaire, Reliability, Validity, Greek translation

## Abstract

**Background:**

Job satisfaction is fundamental to employee well-being and successful operation of an organization. The use of effective tools for assessing it is imperative for management research. Our main purpose was to translate and adapt the Job Satisfaction Survey (JSS) questionnaire to the Greek language and to test its psychometric properties.

**Methods:**

The tool was translated into Greek and then back into English by different bilingual translators. The Greek JSS was tested with a sample of 239 employees of various specialties in drug addiction treatment. Confirmatory Factor Analysis (CFA) for validity testing as well as internal consistency analysis for reliability testing was conducted.

**Results:**

The results confirmed that: (a) the translated version is an accurate translation of the original, (b) CFA results indicated that the nine-factor structure model was a great choice; the factor loads were high and ranged from 0.61 to 0.90, and (c) the reliability coefficients were satisfactory (Cronbach’s alpha for eight of the nine dimensions of the Greek JSS scale ranged from 0.62 to 0.87 except for the dimension “Operating procedures” which was 0.48, while Cronbach’s alpha for the total scale was 0.87 and the Gutman Split-Half Coefficient was 0.88).

**Conclusions:**

The findings suggested that the Greek Version of JSS is a valid and reliable tool for measuring job satisfaction in Greece. Further research for assessing its psychometric values in various samples and further analysis for studying its validity and testing its internal and external consistency and coherence might be conducted in the future.

**Electronic supplementary material:**

The online version of this article (10.1186/s40359-018-0241-4) contains supplementary material, which is available to authorized users.

## Background

Job satisfaction is one of the most frequently studied variables in organizational behavior research [1] and an important predictor for wellness in the work environment. It is directly linked to absenteeism and staff turnover, having at the same time a profound impact on the productivity and the effectiveness of the services that an organization provides [2–4]. Meanwhile, since the majority of people spend between one and two-thirds of their time awake in the workplace, it has a major impact on employee psychological wellbeing at home, affecting many aspects of his/her everyday life [1, 5].

The most-used research definition of job satisfaction was given by Locke [6], who defined it as a positive or pleasing emotional state resulting from the appraisal of one’s job or job experiences. According to the above, employees form their attitudes towards their jobs by considering both their feelings and their beliefs. A simple but comprehensive definition was proposed by Spector [1], who designates job satisfaction as the extent to which someone likes (satisfaction) or dislikes (dissatisfaction) his/her job.

Job satisfaction has two dimensions, intrinsic and extrinsic. Intrinsic job satisfaction refers to how people feel about the nature of the job tasks. For example, the ability to develop one’s skills, a sense of autonomy, success, achievement and control. Extrinsic job satisfaction refers to how people feel about different aspects of the work situation that are external to the work itself; for example, salary, relationships with colleagues, promotion opportunities and the quality of the job environment) [7, 8].

Different instruments to measure job satisfaction have been developed. The main types of instruments are: global instruments that aim to assess global job satisfaction without reference to any specific facets (e.g. Job in General Scale-JIG) [9]; multidimensional instruments that refer to the facet approach (e.g. Job Diagnostic Survey–JDS) [10]; scales that may examine both global job satisfaction as well as its dimensions (e.g. Job Satisfaction Survey-JSS) [11]; instruments that measure one specific job satisfaction dimension (e.g. Pay Satisfaction Questionnaire–PSQ) [12]; whereas different instruments have been designed for jobs in general (e.g. Job Descriptive Index-JDI) or for a specific workforce (Emergency Physician Job Satisfaction Scale-EPJS) [13].

Spector constructed the JSS (Job Satisfaction Survey) which is a multidimensional instrument. Although it was originally developed for the social service sector, he argues that it can be used for other sectors as well [11]. It is one of the most frequently used job satisfaction instruments, while many studies about its psychometrical features have been conducted till today [14–16]. Besides, in a study, reviewing the psychometric quality, reliability, validity and reveal responsiveness of 29 job satisfaction instruments, JSS was one of the seven tools that met the defined and validity criteria [13].

The main purpose of the current study was to translate and investigate further the structure and to assess the factorial validity and the internal consistency of the Greek version of JSS. The results of the validity and reliability studies of an instrument assessing job satisfaction are of importance for further theoretical and empirical studies in this particular field.

## Methods

### Participants

The current study was conducted at the Therapy Center for Dependent Individuals (KETHEA), which is the largest rehabilitation and social reintegration network for drug addicts and their families in Greece, in the spring of 2015. Employees of all categories (administrative staff, therapy – prevention staff, education – research staff, part-time trainers and other staff) comprised the sample. Questionnaires were distributed to 341 employees and were completed by 239.

### Instrument

The Job Satisfaction Survey (JSS) has 36 items with nine subscales to assess employee attitudes about their job and its different aspects. Each subscale is assessed with four items, while a total score is computed from all items. Each item is ranked on a 6-point Likert scale ranging from “strongly disagree” to “strongly agree”. The 36 items are written in both directions, so about half of them must be reverse scored. The nine subscales are Pay, Promotion, Supervision, Fringe Benefits (Monetary and nonmonetary fringe benefits), Contingent Rewards (performance-based rewards), Operating Procedures (Operating policies and required rules), Coworkers, Nature of Work, and Communication. Although the JSS was originally developed for use in human service organizations, it is applicable to a wide range of organization types in both public and private sector [11].

Scores on each of the nine-facet subscales, which in turn are based on 4 items each, can range from 4 to 24, and scores for total job satisfaction, which are based on the sum of all 36 items, can range from 36 to 216. JSS has 19 negatively worded items, which must be reversed. A score of 6 representing strongest agreement with a negatively worded item is considered equivalent to a score of 1 representing strongest disagreement on a positively worded item [11].

As far as scoring interpretation is concerned, two approaches, normative and absolute, can be used. The normative approach would compare the target sample to the norms for the sample, which are limited in three ways: first, there is a small number of occupations and organizations represented; second, the norms are not from representative samples; and third, the norms are mainly from North America—Canada and the U.S., which means that these norms are not representative of other countries that are culturally dissimilar to North America. According to the absolute approach, scores with a mean item response (after reverse scoring) of 4 or more represent satisfaction, mean responses of 3 or less represent dissatisfaction, whereas mean scores between 3 and 4, ambivalence. So, as far as the summed scores are concerned, for the 4-item subscales, scores of 4 to 12 represent dissatisfaction, 16 to 24 represent satisfaction, and those between 12 and 16 represent ambivalence. For the 36-item total, the ranges are 36 to 108 for dissatisfaction, 144 to 216 for satisfaction, and between 108 and 144 for ambivalence. If some items are missing, an adjustment must be done, otherwise the score will be too low. The best procedure is to compute the mean score per item for the individual, and substitute that mean for the missing items. An alternative but less accurate procedure is a middle response substitution for each of the missing items, where either 3 and 4 could be used [17].

### Translation procedure

The forward–backward translation, which is the most commonly applied translation process for questionnaires or inventories, was performed [18]. In the first step of the procedure, the original English of the JSS was translated into Greek language by two experienced translators. The assessment of forward translation drafts was performed by two other researchers who were asked to review each translated item independently and choose the most adequate in terms of clarity, common language and cultural diversity.

The second step included retranslation of the agreed Greek text to English language by a researcher who had not previously seen the original version. The backward translation was compared with the original version of the survey, and judgments about the inaccuracies were made by two other researchers. The resulting differences were finally checked by another scientist who made the necessary adjustments.

The final version of the questionnaire was given to 12 volunteer participants (5 male and 7 female) for pilot testing. Each participant was given a brief introduction and requested to complete the Greek version of the questionnaire independently after which each one was interviewed about its clarity and understandability. The participants confirmed that the Greek version of the JSS was coherent and easy to fill in. The Greek and the English versions of the JSS are shown in the Additional file 1.

### Statistical analysis

Descriptive statistics were used. Quantitative variables were expressed as mean values (SD) and qualitative as absolute and relative frequencies. A confirmatory factor analysis (CFA) with maximum likelihood procedure was conducted in order to test how well the dimensions of the JSS fit the data. The variance of the latent constructs was fixed at one during parameter estimation and the factors were allowed to be correlated. The fit of the CFA model was assessed using the chi square (χ2), the comparative fit index (CFI), the goodness of fit index (GFI) and the root mean square error of approximation (RMSEA) [19]. For the CFI and GFI indices, values close to or greater than 0.95 are taken to reflect a good fit to the data [20]. RMSEA values of less than 0.05 indicate a good fit and values as high as 0.08 indicate a reasonable fit [20]. Pearson coefficients were used to explore intercorrelations among subscales. Reliability analysis included Cronbach’s Alpha for internal consistency and Guttman Split-Half coefficient. Statistically significant level was set at .05 and the analysis was conducted using SPSS and AMOS (SPSS, Chicago, IL, USA) Statistical Software.

## Results

### Sample characteristics

Of the 341 questionnaires distributed, 239 were returned fully completed (70.09% response rate). More than 64% of the study participants were female and the majority were aged between 35 and 39 (34.3%) or between 40 and 50 years (45.2%). As far as educational level is concerned, the majority was university graduates, while 38.1% had post-graduate studies. Concerning working position, the majority worked as Therapy – Prevention staff (56.1%), followed by Administrative staff (20.1%), Education – Research staff (12.1%), Part-time Trainers (7.5%) and other staff (4.2%). As regards length of service, 37.2% of study participants had worked from 11 to 15 years, 28% from 6 to 10 years, 13.8% from 16 to 20 years 12.1% from 0 to 5 years, 7.1% from 20 to 25 years, while 1.7% had worked for more than 26 years (Table 1).

**Table 1:** Demographic features of the Sample

Characteristics	Participants (*n* = 239)		
		N	%
Gender	Women	153	64
	Men	86	36
Age (years)	25–29	3	1,3
	30–34	20	8.4
	35–39	82	34.3
	40–49	108	45.2
	> 50	26	10.9
Educational level	Post-graduate studies	91	38.1
	University graduate	85	35.6
	Τechnological Institutions graduate	23	9.6
	2 year Technical School (Post Secondary)	11	4.6
	Upper Secondary Education	23	9.6
	Low Secondary Education	6	2.5
Specialty	Administrative staff	48	20.1
	Therapy – Prevention staff	134	56.1
	Education – Research staff	29	12.1
	Part-time Trainers	18	7.5
	Other Staff	10	4.2
Professional Experience (years)	0–5	29	12.1
	6–10	67	28
	11–15	89	37.2
	16–20	33	13.8
	20–25	17	7.1
	> 26	4	1.7

### Descriptive statistics

The highest mean from the scale was in the dimension of “Nature of work” (Mean = 18.8), while the lowest mean was recorded in the dimension of “Pay” (Mean = 9.5). Mean scores were comparable with the data of the original study of Spector (1985), where the highest mean was in the dimension of “Supervision” (M = 19.9) and the lowest in “Pay” (Mean = 10.5). The general job satisfaction mean in the Greek sample was lower (Mean = 128.3) than that of the American sample (Mean = 133.1) (Table 2).

**Table 2:** Comparative presentation of mean scores and Standard Deviation of JSS in U.S.A. and Greece

	American Sample (*N* = 3067) Spector (1985)		Greek Sample (*N* = 239)	
Subscale	Mean	S.D.	Mean	S.D.
Pay	10.5	5.1	9.5	3.6
Promotion	11.5	5.1	10.1	3.9
Supervision	19.9	4.6	18.6	4.6
Fringe Benefits	13.1	5.0	11.6	4.6
Contigent Rewards	13.4	5.1	14.0	4.1
Operating Procedures	12.5	4.6	13.1	3.6
Coworkers	18.8	3.7	18.1	3.3
Nature of Work	19.2	4.4	18.8	3.3
Communication	14.0	5.0	14.6	4.3
Total Satisfaction	133.1	27.9	128.3	20.5

### Statistical analysis of validity

The CFA assessed the fit of the nine-factor structure and the model fitted the data well as defined from the RMSEA, CFI and GFI values that were equal to 0.055, 0.951 and 0.946, respectively. None of the item cross loadings exceeded the item loadings on the intended latent construct. Factor loadings were high and ranged from 0.61 to 0.90 indicating a strong association between the latent factors and their respective items.

Table 3 indicates that intercorrelations among subscales range between 0.13 and 0.56. More specifically, correlations were high in three cases, while there were also six medium and six low intercorrelations. The above finding resembles Spector’ s results [11], which also reported low to medium intercorrelations among subscales, ranging from .11 to .59.

**Table 3:** Pearson Correlations among JSS Subscales in Greek Data

	Pay	Promotion	Supervision	Fringe Benefits	Contingent Rewards	Operating Procedures	Coworkers	Nature of Work
Pay								
Promotion	.32*							
Supervision	−.08	.26*						
Fringe Benefits	.48*	.32*	−.01					
Contingent Rewards	.51*	.42*	.37*	.38*				
Operating Procedures	.29*	.10	.15*	.14*	.40*			
Coworkers	−.07	.13*	.56*	−.06	.28*	.14*		
Nature of Work	.01	.23*	.34*	−.07	.32*	.17*	.39*	
Communication	.11	.26*	.47*	.14*	.52*	.30*	.43*	.39*

*p < 0.050

### Statistical analysis of reliability

Cronbach alpha coefficient for each dimension of the Greek JSS scale ranged from 0.62 to 0.87 except in the dimension “Operating procedures” where it was 0.48. Overall, the reliability estimate for the total scale was 0.87 for the thirty-six items of the adapted scale. The comparative presentation of Cronbach’s alpha coefficients between the American sample of Spector’s original study and the Greek sample of the current study is shown in Table 4.

**Table 4:** Comparative presentation of internal consistency coefficients of JSS in U.S.A. and Greece

	American Sample Spector (1985)		Greek Sample	
Subscale	N	Cronbach Alpha	N	Cronbach Alpha
Pay	2870	0.75	239	0.62
Promotion	2870	0.73	239	0.67
Supervision	2870	0.82	239	0.87
Fringe Benefits	2870	0.73	239	0.73
Contigent Rewards	2870	0.76	239	0.71
Operating Procedures	2870	0.62	239	0.48
Coworkers	2870	0.60	239	0.67
Nature of Work	2870	0.78	239	0.74
Communication	2870	0.71	239	0.71
Total Satisfaction	2870	0.91	239	0.87

Split-half reliability was also done by dividing the measure into two halves; (a) consisted of first 18 items and (b) consisted of the remaining 18 items of the scale. The findings showed that JSS had good split-half reliability as assessed through Guttman Split-Half Coefficient (.876) (Table 5).

**Table 5:** Split-Half reliability analysis

		Split-Half Reliability	
Cronbach’s Alpha	Part 1	Value	.747
		N of Items	18(a)^a^
	Part 2	Value	.776
		N of Items	18(b)^a^
		Total N of Items	36
Correlation Between Forms			.781
Spearman-Brown Coefficient		Equal Length	.877
		Unequal Length	.877
Guttman Split-Half Coefficient			.876

^a^a. The items are: JSS 1, JSS 2, JSS 3, JSS 4, JSS 5, JSS 6, JSS 7, JSS 8, JSS 9, JSS 10, JSS 11, JSS 12, JSS 13, JSS 14, JSS 15, JSS 16, JSS 17, JSS 18

^a^b. The items are: JSS 19, JSS 20, JSS 21, JSS 22, JSS 23, JSS 24, JSS 25, JSS 26, JSS 27, JSS 28, JSS 29, JSS 30, JSS 31, JSS 32, JSS 33, JSS 34, JSS 35, JSS 36

## Discussion

The purpose of the current study was the translation of the JSS into Greek and the examination of its psychometric properties. The JSS is a widely used tool which has been translated in more than nineteen languages [17]. Cultural variability could seriously affect the design and adaptation of a questionnaire [21]. Previous studies’ results, examining the psychometric properties of JSS in different countries and cultural contexts, support Spector and Wimalasiri’s [22] claim that cultural differences are found to be underlying the JSS structure. In some cases like Turkey [15] and Pakistan [16], factor structures of the translated versions were found to be similar to the original survey. On the other hand, studies in other countries led to different structure models. In a study in Iranian population, seven factors were identified [23] in a study on Malaysian employees, eight, four and three factor analysis solutions were reported [24], in a study in Uganda, a four-factor solution was the best model [25], while in the Ukraine the best model came out to be a three-facet model [14]. Concerning the validity of the Greek version, the results of CFA of our study indicated that the nine-factor structural model of the JSS was well-adapted and consistent with the original version of the tool. From the nine facets of JSS, nature of work, supervision and promotion are the subscales that fit well both in cases that validation process in different countries revealed consistency with the original nine facet version [15, 16] and in cases that some of the JSS subscales poorly explained some of the tool aspects or led to different factorial structure [14, 23]. On the contrary, the structure of the other JSS factors seems to be strongly affected from cross-cultural differences that in many cases lead to factor variation.

As far as reliability and internal consistency are concerned, values show that the scale items are consistent with one another in as many as eight of the nine subscales. The measures whose Cronbach’s Alpha exceeds 0.60 are considered to be the reliable ones [26]. In the current study, the total scale and all subscales but one were over 0.60. This finding shows that there is adequate internal consistency for the JSS total and for its subscales except the “operating procedures” facet. Internal consistency values for the rest 8 subscales and the total scale were consistent with Spector’s original study findings (Table 4). Moreover, according to the findings, the Greek version of the JSS has good split-half reliability (0.88).

### Limitations

Like any other research in social sciences, the current study also has certain limitations. First, the sample was relatively small. Second, the data was collected only from employees working in drug addiction treatment; therefore, it can’t be generalized for all employees providing healthcare services. Third, almost half of KETHEA employees took part in the study, as they were the only ones available. Therefore, the extent to which results may be generalized is limited and should be treated cautiously. Forth, we did not estimate the convergent validity by comparing JSS with a similar tool and the discriminant validity by comparing JSS with a tool designed to measure a different concept. Finally, the use of the test retested the reliability method to examine the degree to which the results are consistent over time, as it was not feasible in the current field of research.

## Conclusions

Future studies might apply the JSS Greek version to other samples that will include a diverse population from both the public and private sector and beyond social services, in order to establish valuable results and determine the norms of the scale for a wider range of professionals and organizations. In addition, future researchers may examine the criterion, convergent and discriminant validity of the JSS. However, despite the above limitations, it is our hope that the Greek JSS will be used in the future in studies related to job satisfaction.

## Additional file

Additional file 1: Simultaneous presentation of the English and Greek version of JSS. (DOCX 23 kb)

## Abbreviations

CFA: Confirmatory Factor Analysis
CFI: Comparative Fit Index
EPJS: Emergency Physician Job Satisfaction Scale
GFI: Goodness of Fit Index
JDI: Job Descriptive Index
JDS: Job Diagnostic Survey
JIG: Job in General Scale
JSS: Job Satisfaction Survey
KETHEA: Therapy Center for Dependent Individuals
PSQ: Pay Satisfaction Questionnaire
RMSEA: Root Mean Square Error of Approximation
