# Supplementary material for: Validity and reliability of the Greek translation of the Job Satisfaction Survey (JSS)
Source: BMC Psychol. 2018 Jun 8;6:27. doi: 10.1186/s40359-018-0241-4 (PMC5994062; doi:10.1186/s40359-018-0241-4)
Supplement: Supplementary file 1 — Simultaneous presentation of the English and Greek version of JSS. (DOCX 23 kb) [file 40359_2018_241_MOESM1_ESM.docx]

**APPENDIX**

**Simultaneous presentation of the English and Greek version of JSS.**

| Stem question: "Please circle the one number for each question that comes closest to reflecting your opinion about it" Response on a 6-point Likert scale: 1 (Disagree very much) to 6 (Agree very much) | |
| --- | --- |
| 1. I feel I am being paid a fair amount for the work I do. | 1. Πιστεύω ότι αμείβομαι δίκαια για την εργασία που κάνω |
| 2. There is really too little chance for promotion on my job. | 2. Υπάρχουν πολύ λίγες ευκαιρίες για προαγωγή στην εργασία μου |
| 3. My supervisor is quite competent in doing his/her job. | 3. Ο/Η προϊστάμενος/η μου είναι πολύ ικανός/η στη δουλειά του/της. |
| 4 . I am not satisfied with the benefits I receive. | 4. Δεν είμαι ικανοποιημένος από τις πρόσθετες παροχές (ασφαλιστική κάλυψη, άδειες μετ’ αποδοχών, συνταξιοδοτικές προοπτικές) που λαμβάνω, πέραν του μισθού |
| 5. When I do a good job, I receive the recognition for it that I should receive. | 5. Όταν κάνω καλά τη δουλειά μου, λαμβάνω την αναγνώριση που θα έπρεπε |
| 6. Many of our rules and procedures make doing a good job difficult. | 6. Πολλοί από τους κανόνες και τις διαδικασίες που ακολουθούνται στον οργανισμό με δυσκολεύουν στο να κάνω τη δουλειά μου σωστά |
| 7. I like the people I work with. | 7. Συμπαθώ τους ανθρώπους μαζί με τους οποίους δουλεύω |
| 8. I sometimes feel my job is meaningless. | 8. Κάποιες φορές αισθάνομαι ότι η εργασία μου δεν έχει νόημα |
| 9. Communications seem good within this organization. | 9. Η επικοινωνία μέσα στον οργανισμό φαίνεται να είναι καλή |
| 10. Raises are too few and far between. | 10. Οι αυξήσεις του μισθού είναι πολύ σπάνιες |
| 11. Those who do well on the job stand a fair chance of being promoted. | 11. Αυτοί που κάνουν καλά την δουλειά τους έχουν αρκετές πιθανότητες προαγωγής |
| 12. My supervisor is unfair to me. | 12. Ο/Η προϊστάμενος/η μου είναι άδικος/η μαζί μου |
| 13. The benefits we receive are as good as most other organizations offer. | 13. Οι πρόσθετες παροχές- πέραν του μισθού που λαμβάνουμε από την εργασία είναι εξίσου καλές με αυτές που προσφέρουν άλλοι οργανισμοί |
| 14. I do not feel that the work I do is appreciated. | 14. Θεωρώ πως η δουλειά μου δεν εκτιμάται |
| 15. My efforts to do a good job are seldom blocked by red tape. | 15. Οι προσπάθειές μου να κάνω καλά τη δουλειά μου σπάνια εμποδίζονται από τη γραφειοκρατία |
| 16. I find I have to work harder at my job because of the incompetence of people I work with. | 16. Θεωρώ ότι πρέπει να εργάζομαι σκληρότερα εξαιτίας  της ανικανότητας των συναδέλφων μου |
| 17. I like doing the things I do at work. | 17. Μου αρέσουν τα πράγματα που κάνω στη δουλειά μου |
| 18. The goals of this organization are not clear to me. | 18. Οι στόχοι του οργανισμού στον οποίο εργάζομαι δεν μου είναι ξεκάθαροι |
| 19. I feel unappreciated by the organization when I think about what they pay me. | 19. Όταν σκέφτομαι την αμοιβή μου αισθάνομαι ότι δεν εκτιμάται η εργασία μου από τον οργανισμό |
| 20. People get ahead as fast here as they do in other places. | 20. Οι εργαζόμενοι εδώ προάγονται τόσο γρήγορα όσο και σε άλλες δουλειές |
| 21. My supervisor shows too little interest in the feelings of subordinates. | 21. Ο/η προϊστάμενος/η μου δείχνει ελάχιστο ενδιαφέρον για το πώς αισθάνονται οι υφιστάμενοί του |
| 22. The benefit package we have is equitable. | 22. Το πακέτο πρόσθετων παροχών - πέραν του μισθού που λαμβάνουμε στα πλαίσια της εργασίας είναι δίκαιο |
| 23. There are few rewards for those who work here. | 23. Υπάρχουν λίγες ανταμοιβές για αυτούς που εργάζονται εδώ |
| 24. I have too much to do at work. | 24. Έχω περισσότερο φόρτο εργασίας απ’ ότι θα έπρεπε |
| 25. I enjoy my coworkers. | 25. Περνώ καλά με τους συναδέλφους μου |
| 26. I often feel that I do not know what is going on with the organization. | 26. Συχνά αισθάνομαι ότι δεν γνωρίζω τι συμβαίνει στον οργανισμό στον οποίο εργάζομαι |
| 27. I feel a sense of pride in doing my job. | 27. Νιώθω μια αίσθηση υπερηφάνειας για τη δουλειά που κάνω |
| 28. I feel satisfied with my chances for salary increases. | 28. Αισθάνομαι ικανοποιημένος από τις ευκαιρίες μισθολογικών αυξήσεων που μου παρέχονται |
| 29. There are benefits we do not have which we should have. | 29. Υπάρχουν πρόσθετες παροχές πέραν του μισθού που θα έπρεπε να λαμβάνουμε αλλά αυτό δεν συμβαίνει |
| 30. I like my supervisor. | 30. Συμπαθώ τον/την προϊστάμενο/η μου |
| 31. I have too much paperwork. | 31. Έχω περισσότερη γραφειοκρατική δουλειά από ότι θα ‘πρεπε |
| 32. I don't feel my efforts are rewarded the way they should be. | 32. Θεωρώ ότι οι προσπάθειές μου δεν ανταμείβονται όπως θα ’πρεπε |
| 33. I am satisfied with my chances for promotion. | 33. Είμαι ικανοποιημένος από τις ευκαιρίες προαγωγής που  μου παρέχονται |
| 34. There is too much bickering and fighting at work. | 34. Υπάρχουν πολλοί διαπληκτισμοί και διαμάχες στη δουλειά |
| 35. My job is enjoyable. | 35. Η εργασία μου είναι ευχάριστη |
| 36. Work assignments are not fully explained. | 36. Τα εργασιακά καθήκοντα ανατίθενται χωρίς να επεξηγούνται πλήρως. |
